# Radio-pathomic maps of glioblastoma identify phenotypes of non-enhancing tumor infiltration associated with bevacizumab treatment response

**DOI:** 10.1007/s11060-024-04593-7

**Published:** 2024-02-19

**Authors:** Samuel A. Bobholz, Alisha Hoefs, Jordyn Hamburger, Allison K. Lowman, Aleksandra Winiarz, Savannah R. Duenweg, Fitzgerald Kyereme, Jennifer Connelly, Dylan Coss, Max Krucoff, Anjishnu Banerjee, Peter S. LaViolette

**Affiliations:** 1 Department of Radiology, Medical College of Wisconsin, 8701 Watertown Plank Rd, 53226 Milwaukee, WI USA; 2 Department of Biophysics, Medical College of Wisconsin, Milwaukee, WI USA; 3 Department of Neurology, Medical College of Wisconsin, Milwaukee, WI USA; 4 Department of Pathology, Medical College of Wisconsin, Milwaukee, WI USA; 5 Department of Neurosurgery, Medical College of Wisconsin, Milwaukee, WI USA; 6 Department of Biostatistics, Medical College of Wisconsin, Milwaukee, WI USA; 7 Department of Biomedical Engineering, Medical College of Wisconsin, Milwaukee, WI USA

**Keywords:** MRI, Glioblastoma, Glioma, Radio-pathomics, Radiomics, Bevacizumab, Radio-pathomic mapping

## Abstract

**Background:**

Autopsy-based radio-pathomic maps of glioma pathology have shown substantial promise inidentifying areas of non-enhancing tumor presence, which may be able to differentiate subsets of patients that respond favorably to treatments such as bevacizumab that have shown mixed efficacy evidence. We tested the hypthesis that phenotypes of non-enhancing tumor fronts can distinguish between glioblastoma patients that will respond favorably to bevacizumab and will visually capture treatment response.

**Methods:**

T1, T1C, FLAIR, and ADC images were used to generate radio-pathomic maps of tumor characteristics for 79 pre-treatment patients with a primary GBM or high-grade IDH1-mutant astrocytoma for this study. Novel phenotyping (hypercellular, hypocellular, hybrid, or well-circumscribed front) of the non-enhancing tumor front was performed on each case. Kaplan Meier analyses were then used to assess differences in survival and bevacizumab efficacy between phenotypes. Phenotype compartment segmentations generated longitudinally for a subset of 26 patients over the course of bevacizumab treatment, where a mixed effect model was used to detect longitudinal changes.

**Results:**

Well-Circumscribed patients showed significant/trending increases in survival compared to Hypercellular Front (HR = 2.0, *p* = 0.05), Hypocellular Front (HR = 2.02, *p* = 0.03), and Hybrid Front tumors (HR = 1.75, *p* = 0.09). Only patients with hypocellular or hybrid fronts showed significant survival benefits from bevacizumab treatment (HR = 2.35, *p* = 0.02; and HR = 2.45, *p* = 0.03, respectively). Hypocellular volumes decreased by an average 50.52 mm^3^ per day of bevacizumab treatment (*p* = 0.002).

**Conclusion:**

Patients with a hypocellular tumor front identified by radio-pathomic maps showed improved treatment efficacy when treated with bevacizumab, and reducing hypocellular volumes over the course of treatment may indicate treatment response.

## Introduction

Glioblastoma (GBM) is an aggressive, heterogenous, diffuse glioma with one- and five-year survival rates of 41% and 5%, respectively [1, 2]. Clinical standard of care for glioblastoma patients begins with maximal safe resection of the primary tumor mass, followed by radiation and concomitant temozolomide [3, 4]. Once the tumor recurs, treatment paths deviate based on clinical monitoring of the disease and patient-related factors. At recurrence, salvage therapy can include angiogenic drugs [5, 6], immunotherapy [7–9], tumor treating fields [10, 11], and repeat surgery or radiation [12, 13]. Bevacizumab (Bev) is the most common form of anti-angiogenic agent in the United States, which acts by binding to and inhibiting vascular endothelial growth factor A (VEGF-A) and thus hindering the development of tumor vasculature [14]. Clinical trials have been inconclusive for suggesting an overall survival improvement related to bevacizumab use, thus it is more commonly prescribed to improve quality of life for patients in later stages of the disease [14–16].

Further complicating the use of bevacizumab is the effect of angiogenic agents on traditional imaging signatures for tumor presence and treatment response. Contrast-enhanced T1-weighted images (T1C) are used to define the primary tumor mass for both surgical resection and treatment-response monitoring, which capitalizes on leaky tumor vessel formation to selectively highlight the tumor mass [17]. However, it is known that glial tumor invasion occurs well-beyond the contrast-enhancing margin, particularly in later stages of the disease [18, 19]. Anti-angiogenic agents compound this issue by preventing the tumor from forming new vasculature, often creating the appearance of halting tumor growth but potentially failing to address non-enhancing tumor progression [20–22]. Other imaging signatures such as T2-weighted fluid attenuated inversion recovery (FLAIR) hyperintensity and low apparent diffusion coefficient (ADC) calculated from diffusion-weighted imaging can be used to inform clinicians about potential tumor invasion and edemic tissue in the areas surrounding the primary tumor mass, but these signatures show less pronounced relationships with pathological tumor presence in heterogenous, high-grade tumors such as GBMs [23–25]. Therefore, improvements in non-invasive tumor tracking are critical to monitoring the presence of non-enhancing tumor and identifying treatment response, particularly in later stages of disease where radiation treatment effects and anti-angiogenic agents cloud traditional MRI interpretation.

Studies looking to expand the treatable margin for tumors have turned to advanced imaging techniques to address gaps in conventional imaging. Novel acquisitions such as MR spectroscopy [26, 27] and amide proton transfer-weighted chemical exchange saturation transfer (APT-CEST) imaging [28, 29] have targeted changes in cellular metabolism that predate angiogenesis, allowing for earlier detection of cancer development. Machine learning and deep learning studies using biopsy tissue as ground truth have exploited the increased computational efficiency of modern hardware to extract deep textural features from conventional imaging to improve the detection of occult tumor invasion, though these studies are limited to the scope and magnitude of surgically resectable tissue [30, 31]. In recent studies, large format autopsy tissue from glioma patients have been aligned to MR images to develop a radio-pathomic mapping tool that allows for non-invasive detection of cell density, extracellular fluid density, cytoplasm density, and tumor probability [23, 32]. By using a pathologically rich, post-treatment ground truth sampled beyond the presence of traditional imaging signatures, this model can detect previously invisible areas of invasion and distinguishes between areas of tumor and treatment effect. Furthermore, by using traditionally acquired MR images (T1, T1C, FLAIR, ADC), the model can detect non-enhancing tumor presence without extending scan time beyond traditionally acquired images, and it can be retrospectively applied to nearly any imaging session for recent patients treated with brain tumors, which allows for mapping of non-angiogenic tumor development across timepoints.

This study sought to use radio-pathomic maps of tumor cell density to develop a phenotyping system for patterns of non-enhancing tumor presence. These phenotypes were then used to assess differences in both prognosis and bevacizumab treatment response, identifying a subset of patients that selectively respond to angiogenic therapy. This tumor front pattern was then assessed longitudinally to determine if treatment response could be visualized using the radio-pathomic maps. Together, this study tested the hypothesis that radio-pathomic mapping phenotypes of non-enhancing tumor pathology predict and depict bevacizumab treatment response.

## Methods

### Patient population

This study was approved by the Institutional Review board of Medical College of Wisconsin. The primary dataset for this study consisted of a total of 79 patients (age = 61.51 ± 13.05, 40 male, 39 female), diagnosed with a primary glioblastoma or IDH1-mutant astrocytoma with histological glioblastoma features, in accordance with the 2021 WHO brain tumor classification standards [33]. A supplemental dataset of 26 patients diagnosed with a primary glioblastoma with longitudinal imaging during Bev treatment was also used to assess volumetric changes in tumor compartments in response to Bev use.

### MR imaging acquisition and preprocessing

Clinical imaging was collected from each patient’s pre-surgical MRI for inclusion in this study. Pre and post-contrast T1-weighted images (T1, T1 + C), T2-weighted fluid attenuated inversion recovery (FLAIR) images, and apparent diffusion images calculated from diffusion weighted imaging acted as the input for radio-pathomic map generation. Images were preprocessed following the standard used for model generation, which involved alignment of all images to the FLAIR image by using SPM12’s co-registration tools [34, 35], as well as intensity normalization by dividing each voxel by the whole brain intensity standard deviation for each non-quantitative image (T1, T1 + C, FLAIR). For the longitudinal dataset, images were acquired from each patient near the start and end of Bev treatment, as well as approximately every six months during treatment when available. Each patient’s FLAIR image was then aligned to the pre-Bev scan, and cross-sectional alignment and normalization were conducted using the same methodology as the pre-surgical scans.

### Cell density and extracellular fluid mapping

Processed MR images for each patient at each timepoint were then used to generate radio-pathomic maps of cell density and extracellular fluid using a previously developed algorithm [23, 32]. Briefly, large format autopsy samples were collected from areas of suspected tumor and non-tumor and aligned to clinical MRI near death [23, 36–38]. Bagging forest algorithms were used to predict computed features of the pathology including cell density (in cells/mm^2^), ECF density, and cytoplasm density using 5 by 5 voxel tiles from the T1, T1 + C, FLAIR, and ADC images as input, providing voxel-wise maps of tissue characteristics previously only available via biopsy. These maps were trained on 43 patients and tested on 22 held-out patients, showing high accuracy on internal test data and impressive generalizability to external data. The maps were further converted to tumor probability maps via an additional algorithm in the prior manuscript [32], but for this current study only cell density and ECF maps are used. Radio-pathomic maps were generated for each imaging acquisition included in this study using this pre-trained model using a local Matlab toolbox, producing both maps in approximately 10 min per session. Each map was visually inspected to ensure sufficient quality predictions for qualitative annotation and segmentations.

### Phenotyping

**Fig. 1:**
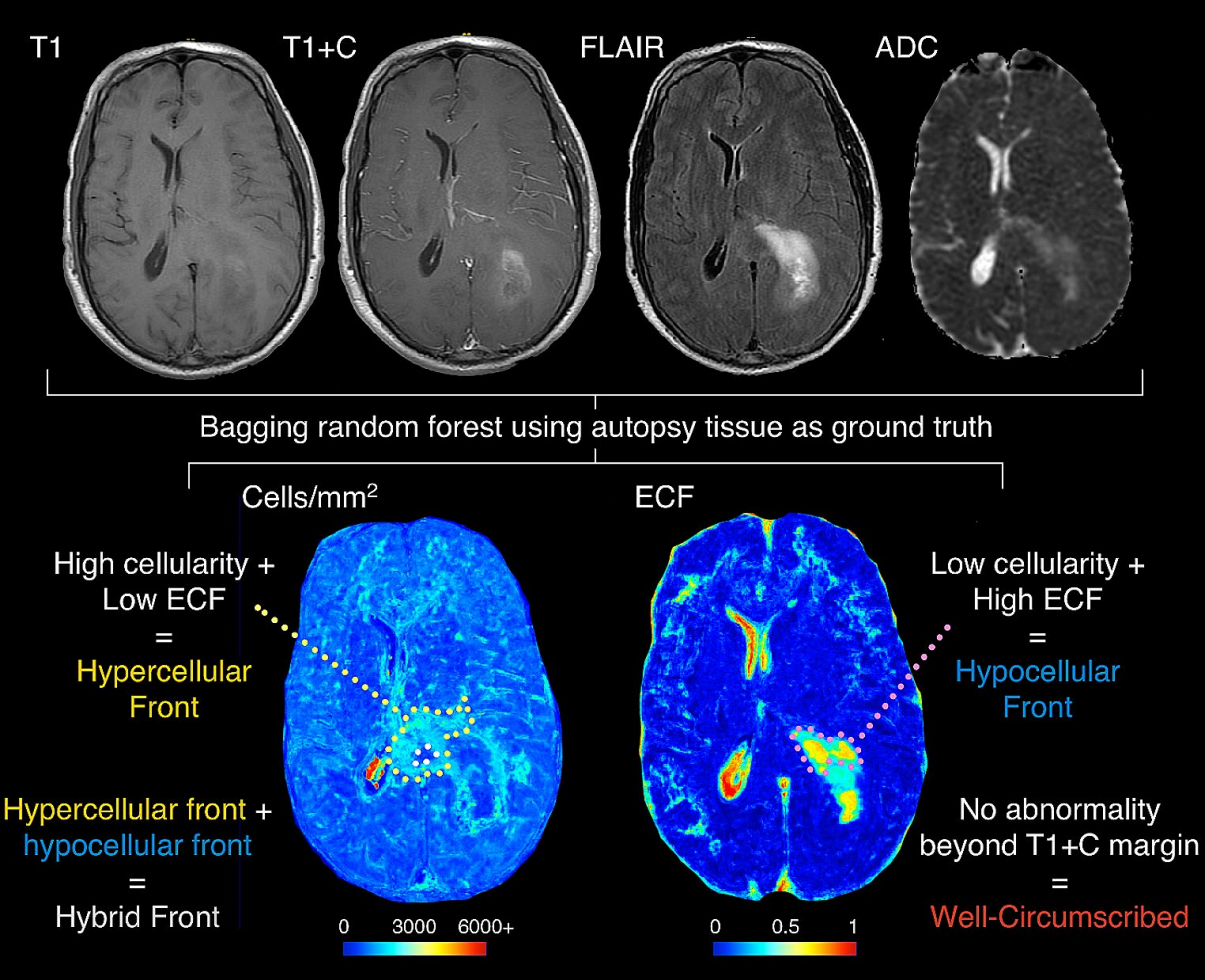
Overview of the study methodology. T1, T1 + C, FLAIR, and ADC images were used to generate radio-pathomic maps of cell density (in cells/mm^2^) and extracellular fluid density (ECF, ranging from 0 to 1). These maps were then graded based on the presence of pathological abnormalities occurring outside the contrast enhancing region where hypercellular front patients exhibited high cell density and low ECF outside contrast, hypocellular front patients exhibited low cell density and high ECF outside contrast, hybrid front patients showed both hypercellular and hypocellular front regions (as seen in this example), and well-circumscribed patients showed no abnormal pathology beyond contrast enhancement

An overview of the tumor phenotyping process is presented in Fig. 1. Subjects were grouped into phenotypes based on their non-enhancing tumor presence on cell density and ECF maps. The rationale for this system was based on the non-enhancing tumor margin being thought to consist primarily of areas of active infiltrative tumor mixed with regions of necrosis and edema, which are readily identifiable characteristics on the cell density and ECF maps trained on autopsy tissue collected from supramarginal tumor regions. Phenotyping was manually conducted, blinded to all information besides the patient’s clinical imaging and radio-pathomic maps. The phenotypes analyzed were selected to characterize non-enhancing pathology surrounding the primary tumor mass, and included: (1) Hypercellular Front, characterized by the presence of high cell density and low ECF presence beyond the contrast-enhancing margin, (2) hypocellular front, characterized by the presence of low cell density and high ECF presence beyond contrast-enhancement (hypothesized to denote areas of edema/necrosis), (3) hybrid front, where areas of both hypercellular and hypocellular fronts coexist within the same patient, and (4) well-circumscribed, which denotes patients with no abnormal pathology beyond the contrast-enhancing region.

### Assessment of bevacizumab response

A Kaplan Meier analysis was used to determine differences in overall survival between phenotypes. Additional Kaplan Meier analyses were used to differentiate phenotypes that showed significant survival benefit in the pre-surgical dataset. Models were fit separately for each phenotype, testing the effect of bevacizumab treatment on overall patient survival within each group. As all patients in the dataset succumbed to the disease, no datapoints were censored for analysis.

### Longitudinal compartmental assessment

To visualize the effects of Bev treatment on tumor front components, manual annotations were drawn for hypercellular and hypocellular areas on the cell density and ECF maps for the longitudinally imaged dataset. Areas of both high cell density and low ECF outside of contrast enhancement were included for hypercellular annotations, and areas of both low cell density and high ECF outside of contrast enhancement were included for the hypocellular annotations. The volume of each region of interest was then computed for each time point. A mixed effect model was used to measure the change in volumes over the course of treatment, including subject as a random effect to account for repeated measures. For all statistical analyses, an alpha of 0.05 was used to determine significance.

## Results

### Assessment of bevacizumab response

**Fig. 2:**
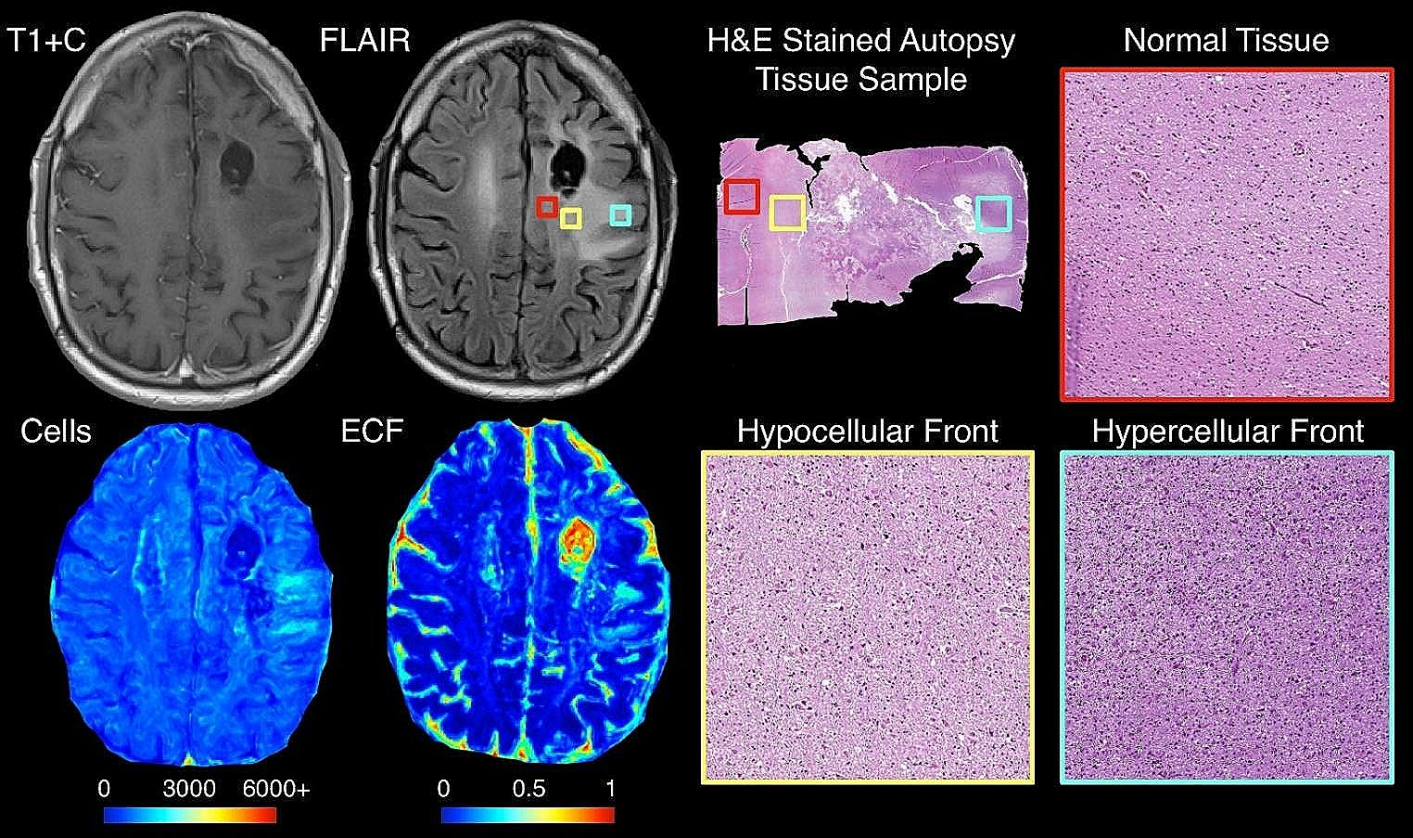
Example imaging and radio-pathomic maps for a hybrid front primary glioblastoma with aligned histology from each front component region. Histological analysis confirmed that each region indicated true areas of hyper- and hypocellularity, respectively, indicating that these phenotypes represent true non-enhancing pathological tumor invasion

**Fig. 3:**
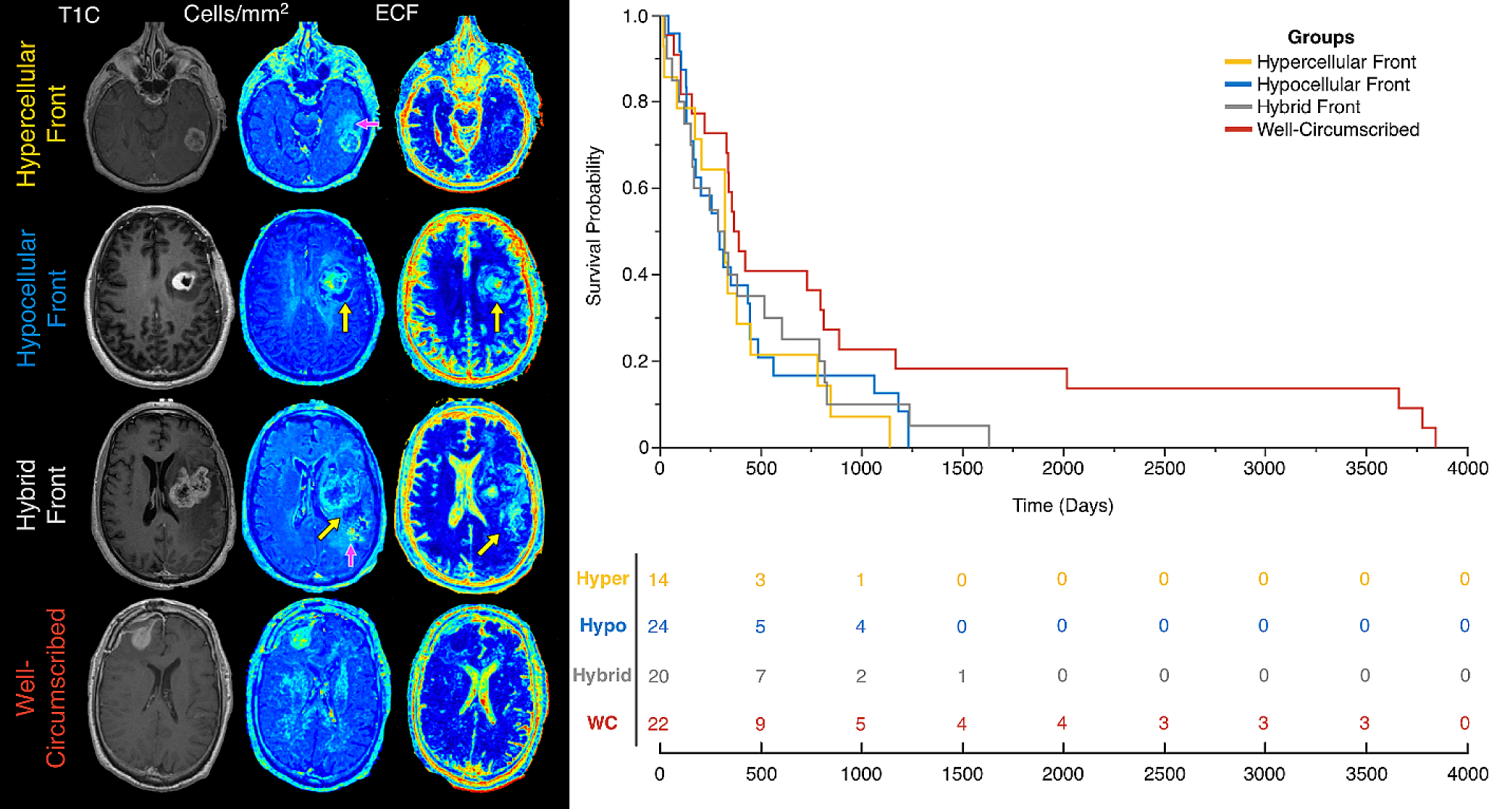
(left) Examples of each phenotype observed in the presurgical dataset. (right) Kaplan-Meier curves for overall survival within each phenotype. Well-circumscribed patients showed trending-to-significant longer overall survival than hypercellular (HR = 2.0, *p* = 0.06), hypocellular, (HR = 2.02, *p* = 0.03), and hybrid (HR = 1.75, *p* = 0.09) phenotypes

**Fig. 4:**
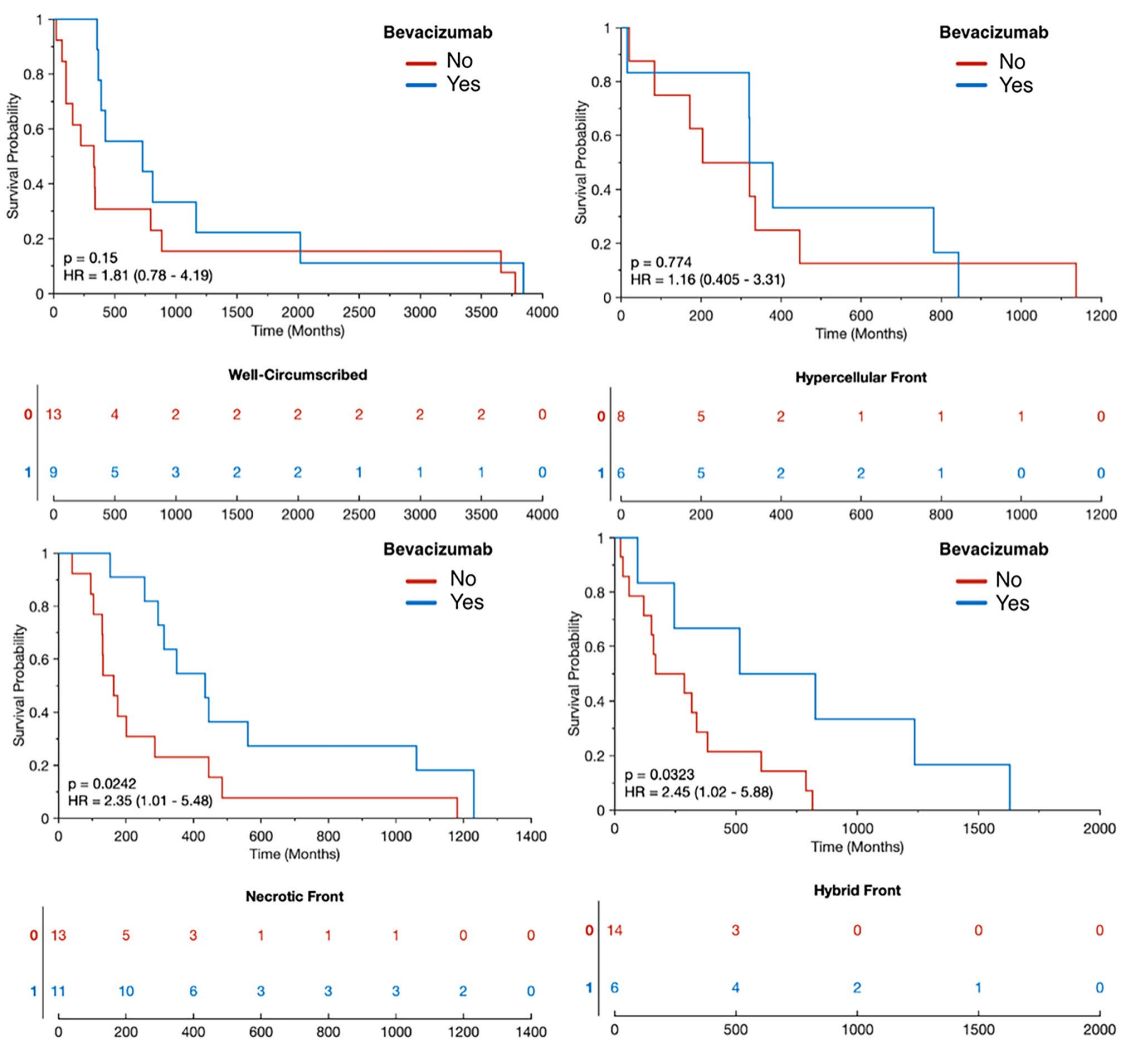
Survival differences between patients who have and have not received bevacizumab within each phenotype. Only patients with a hypocellular component to their tumor front demonstrated an overall survival benefit (hypocellular front HR = 2.35, *p* = 0.024), hybrid front HR = 2.45, 0.032)

Figure 2 shows an example of a hybrid front subject from our autopsy dataset with corresponding autopsy pathology, confirming that areas of predicted hypercellular and hypocellular front correspond to their expected pathological features on histological tissue samples. Areas of hypercellular front show increased nuclear density on histology, areas of hypocellular front show reduced nuclear density and increased ECF presence, and both fronts are readily distinguishable from normal appearing tissue from beyond the tumor front. Figure 3 shows examples of each tumor phenotype, along with their respective Kaplan-Meier survival curves. Well-circumscribed patients showed trending-to-longer survival times compared to other phenotypes (hypocellular front: HR = 2.02, *p* = 0.03; hypercellular front: HR = 2.0, *p* = 0.06; hybrid front: HR = 1.75, *p* = 0.09), with hypocellular front tumors showing the shortest overall survival times. Kaplan Meier curves for patients who have and have not received bevacizumab therapy, split by phenotype, are presented in Fig. 4. No survival benefit was seen for bevacizumab therapy within the hypercellular front group (HR = 1.16, *p* = 0.77) or well-circumscribed group (HR = 1.81, *p* = 0.15). Survival benefit was however observed for both the hypocellular front group (HR = 2.35, *p* = 0.024) and the hybrid front group (HR = 2.45, *p* = 0.032), both phenotypes containing hypocellular presence.

### Longitudinal compartmental assessment

**Fig. 5:**
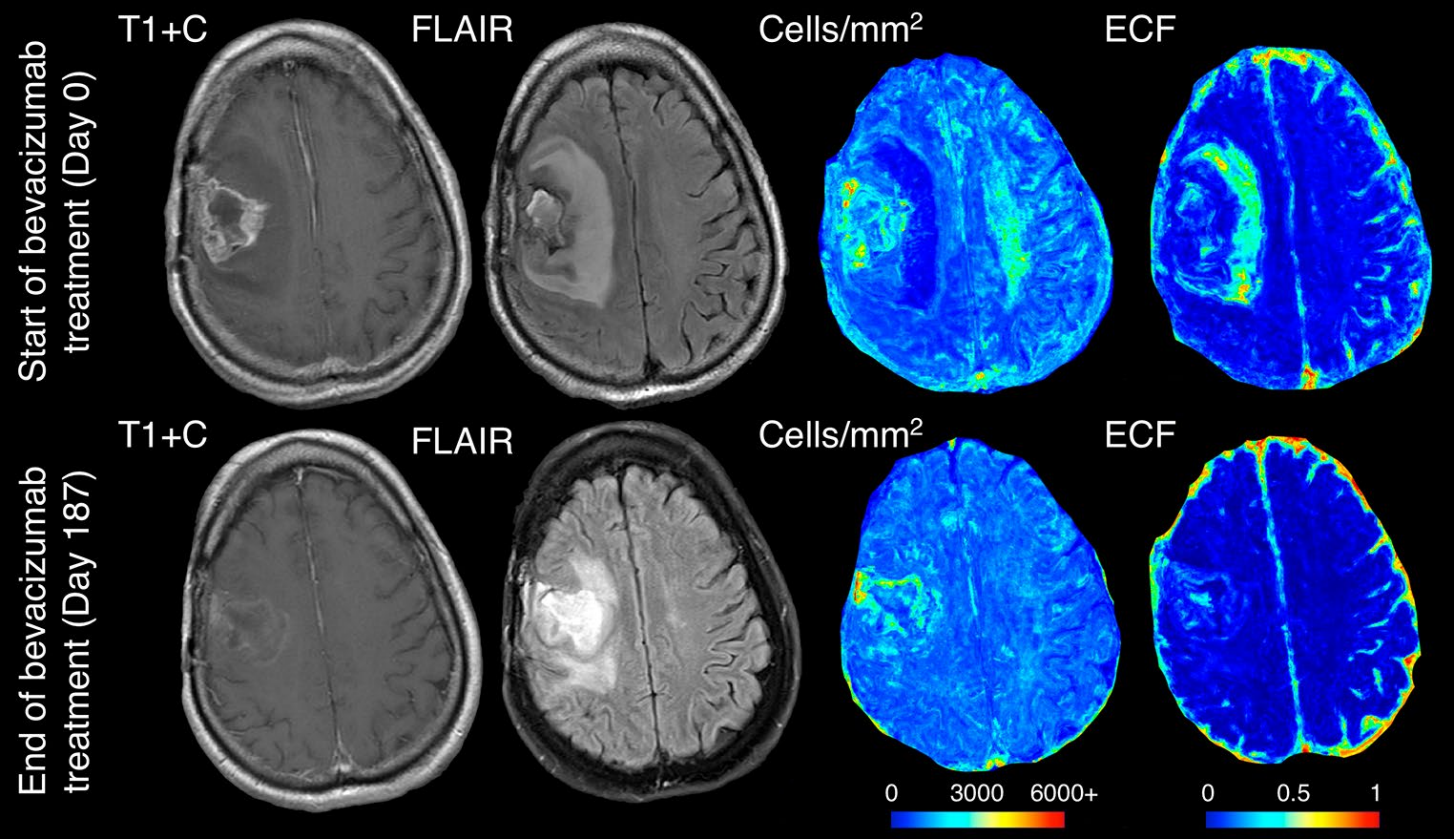
Example imaging and radio-pathomic maps for a hybrid front primary glioblastoma patient before and after bevacizumab treatment, showing a near total reduction in hypocellular component volume over the course of treatment

The mixed effect model assessing hypocellular volumes over time found a decrease in size over the course of bevacizumab treatment (B=-50.51 mm^3^/day, *p* = 0.002). No change was observed for hypercellular volumes over the course of treatment (*p* = 0.357). Figure 5 shows a representative example subject that exhibits the reduction in hypocellular volume observed statistically. Prior to bevacizumab administration, the patient shows a large portion of hypocellular front presence beyond contrast enhancement, which is severely reduced in size on follow-up imaging occurring post-bevacizumab administration.

## Discussion

This study used radio-pathomic maps of cell and extra-cellular fluid density to distinguish between non-enhancing tumor front phenotypes that predict bevacizumab treatment response. This system of phenotypes included well-circumscribed patients who showed the longest overall survival estimates and did not respond to bevacizumab treatment, hypercellular front patients who also did not respond to bevacizumab treatment, hypocellular front patients who showed bevacizumab response and shorter overall survival estimates, and hybrid front patients who show signs of both hyper- and hypocellular fronts and showed bevacizumab treatment response. Additionally, we found that hypocellular components of the tumor decrease in volume in response to treatment, with no similar trend observed for hypercellular components, suggesting the treatment may selectively impact these regions. These results suggest that non-invasive radio-pathomic maps of tumor pathology can track non-enhancing tumor activity over the course of bevacizumab treatment and can identify patients that may selectively respond to therapy.

GBM is a pathologically diverse and heterogenous diagnosis, with several molecular and genetic features known to drive differences in patient outcomes. With each prognostic factor comes an angle of tumor biology that can be exploited for treatment development, as well as the potential to identify a group that selectively responds to a known therapy. MGMT methylation status, for example, is known to directly affect the therapeutic benefit from temozolomide therapy and is thus used as a marker to drive clinical decision-making [39, 40]. The phenotypes in this study provide a first-use case for radio-pathomic maps as a tool for selective treatment response identification. Due to their ability to non-invasively identify macroscopic patterns of tumor infiltration beyond traditional imaging signatures, they can elucidate tumoral heterogeneity difficult to capture both with contrast-weighted imaging and sampling-restricted pathological markers. This technique could therefore be used as a screening tool during clinical decision making, particularly post-recurrence where previously administered radiotherapy is known to cloud traditional MRI interpretation. Future research is warranted into the stability of these phenotypes across a patient’s clinical history, particularly following critical points of tumor development such as recurrence. Additionally, studies examining how other standard-of-care treatments affect hypercellular and hypocellular tumor components may reveal how these non-enhancing tumor patterns react to different interventions and provide a more well-rounded perspective on the full breadth of treatment response.

In the development of new therapeutic approaches for GBM and other diffuse gliomas, it is critical to monitor the pathological response of the tumor to treatment. Particularly with anti-angiogenic agents and other treatments known to impact the appearance tumor on imaging, this becomes challenging as the amount of tumor visible using traditional contrast-enhancing volumes does not capture areas of non-angiogenic tumor growth and FLAIR hyperintense regions fail to distinguish between tumor and edema. This in part has likely led to mixed response data in clinical trials and makes it difficult to assess the therapeutic benefit of such treatments. These maps, in addition to identifying a subpopulation that selectively respond to treatment, have also demonstrated efficacy at expanding treatment response to non-enhancing portions of the tumor. In this study, heterogenous front components for hyper- and hypocellular regions of tumor responded differently to bevacizumab treatment, indicating that specific expressions of GBM pathology can be used identified to more precisely observe treatment response characteristics.

While the delineation of tumor phenotypes that selectively respond to bevacizumab treatment is a promising and useful result, future research remains necessary to better understand hypocell density as a signature for treatment response. While hypercell density has an intuitive relationship with tumor presence, where higher cell density likely indicates actively mitotic cellular proliferation, the relationship between hypocell density and active tumor presence remains less immediately interpretable. These areas could indicate tumor-related necrosis following either tumor progression or pre-angiogenic tumor-induced hypoxia, as histological necrosis was seen in samples where hypocellular areas overlapped with sampled autopsy tissue. However, this limited sample could be expounded upon in both basic science research, where whole-brain resection is more feasible, as well as in autopsy-based clinical research using immuno-histochemical and genetic characterization to better elucidate the pathological profile of these areas. Additionally, further pathological and genetic probing may reveal specific mechanisms sensitive to anti-angiogenic therapy that could explain the bevacizumab treatment benefit observed in the results of this study.

### Limitations

While the radio-pathomic maps used to develop our system of phenotypes for this study have shown great promise in identifying non-enhancing tumor areas, the time between imaging and tissue collection at autopsy remains a source of potential error in these predictions. While limited pathological confirmation at surgery has shown promise in identifying tumor presence in untreated GBMs prior to surgery, future research improving upon these models may provide tighter control over the temporal window of the predictive maps. Tissue distortion and shrinking also occurs during the formalin fixation process leading up to autopsy, which can cause distortions between imaging and histology. We control for this during model development by using a non-linear transform-based registration on samples collected using 3D printed apparatuses to ensure structural stability during slicing and fixation, though it is possible that distortions still occur beyond our control. Additionally, the categorical phenotypes were classified via a single rater blinded to all clinical information besides the imaging; therefore, future research is required to assess the stability between these phenotypes both within raters and over time to ensure reliability. Additionally, other systems of phenotypes characterizing other novel aspects of these radio-pathomic maps may reveal more information regarding tumor composition and its relationship with treatment response and should thus be a target for follow-up studies and future research. Lastly, due to the relatively small sample size of this study, future studies replicating these findings across multiple institutions in larger cohorts are essential to ensuring the reproducibility of these findings. Particularly in the case of the longitudinal changes seen during bevacizumab treatment, larger sample sizes and controlled imaging acquisitions will be critical for understanding how these treatment related changes impact prognosis.

## Conclusions

Radio-pathomic maps of glioblastoma pathology identify phenotypes of supramarginal tumor invasion that distinguish patients who will respond favorably to bevacizumab treatment, with the identified hypocellular tumor front component seeing a decrease in volume over the course of treatment. This technique shows promise in aiding clinical decision making regarding late-stage anti-angiogenic agents and improving non-invasive disease tracking and pathological characterization using conventional imaging alone.

## Data Availability

Study data is available upon reasonable request to the corresponding author.

## References

[1] Ostrom QT, Cote DJ, Ascha M (2018). Adult glioma incidence and survival by race or ethnicity in the United States from 2000 to 2014. JAMA Oncol.

[2] Mathew EN, Berry BC, Yang HW et al (2022) Delivering therapeutics to Glioblastoma: overcoming Biological constraints. Int J Mol Sci 23. 10.3390/ijms23031711 10.3390/ijms23031711 PMC8835860 35163633

[3] Stupp R, Hegi ME, Mason WP (2009). Effects of radiotherapy with concomitant and adjuvant temozolomide versus radiotherapy alone on survival in glioblastoma in a randomised phase III study: 5-year analysis of the EORTC-NCIC trial. Lancet Oncol.

[4] Wen PY, Weller M, Lee EQ (2020). Glioblastoma in adults: a Society for Neuro-Oncology (SNO) and European Society of Neuro-Oncology (EANO) consensus review on current management and future directions. Neuro Oncol.

[5] Reardon DA, Brandes AA, Omuro A (2020). Effect of Nivolumab vs Bevacizumab in patients with recurrent glioblastoma: the CheckMate 143 phase 3 Randomized Clinical Trial. JAMA Oncol.

[6] Garcia J, Hurwitz HI, Sandler AB (2020). Bevacizumab (Avastin®) in cancer treatment: a review of 15 years of clinical experience and future outlook. Cancer Treat Rev.

[7] Arrieta VA, Chen AX, Kane JR (2021). ERK1/2 phosphorylation predicts survival following anti-PD-1 immunotherapy in recurrent glioblastoma. Nat Cancer.

[8] Yang F, He Z, Duan H (2021). Synergistic immunotherapy of glioblastoma by dual targeting of IL-6 and CD40. Nat Commun.

[9] Yuan B, Wang G, Tang X (2022). Immunotherapy of glioblastoma: recent advances and future prospects. Hum Vaccin Immunother.

[10] Glas M, Ballo MT, Bomzon Z (2022). The impact of Tumor Treating fields on Glioblastoma progression patterns. Int J Radiation Oncology*Biology*Physics.

[11] Moser JC, Salvador E, Deniz K (2022). The mechanisms of Action of Tumor Treating fields. Cancer Res.

[12] Di Nunno V, Franceschi E, Tosoni A (2020). Treatment of recurrent glioblastoma: state-of-the-art and future perspectives. Expert Rev Anticancer Ther.

[13] Minniti G, Niyazi M, Alongi F (2021). Current status and recent advances in reirradiation of glioblastoma. Radiat Oncol.

[14] Schäfer N, Proescholdt M, Steinbach JP (2018). Quality of life in the GLARIUS trial randomizing bevacizumab/irinotecan versus temozolomide in newly diagnosed, MGMT-nonmethylated glioblastoma. Neuro Oncol.

[15] Nagpal S, Harsh G, Recht L (2011). Bevacizumab improves quality of life in patients with recurrent glioblastoma. Chemother Res Pract.

[16] Gramatzki D, Roth P, Rushing EJ (2018). Bevacizumab may improve quality of life, but not overall survival in glioblastoma: an epidemiological study. Ann Oncol.

[17] Ellingson BM, Wen PY, Cloughesy TF (2018). Evidence and context of use for contrast enhancement as a surrogate of disease burden and treatment response in malignant glioma. Neuro Oncol.

[18] Autry A, Phillips JJ, Maleschlijski S (2017). Characterization of metabolic, Diffusion, and Perfusion properties in GBM: contrast-enhancing versus non-enhancing Tumor. Transl Oncol.

[19] Lasocki A, Gaillard F (2019). Non-contrast-enhancing Tumor: a New Frontier in Glioblastoma Research. Am J Neuroradiol.

[20] Nguyen HS, Milbach N, Hurrell SL (2016). Progressing Bevacizumab-Induced Diffusion Restriction is Associated with Coagulative Necrosis surrounded by viable tumor and decreased overall survival in patients with recurrent glioblastoma. AJNR Am J Neuroradiol.

[21] Ellingson BM, Kim HJ, Woodworth DC (2014). Recurrent glioblastoma treated with bevacizumab: contrast-enhanced T1-weighted subtraction maps improve tumor delineation and aid prediction of survival in a multicenter clinical trial. Radiology.

[22] Galla N, Chiang G, Chakraborty S (2017). Apparent diffusion coefficient changes predict survival after intra-arterial bevacizumab treatment in recurrent glioblastoma. Neuroradiology.

[23] Bobholz SA, Lowman AK, Brehler M (2022). Radio-pathomic maps of cell density identify Brain Tumor Invasion beyond traditional MRI-Defined margins. AJNR Am J Neuroradiol.

[24] Husstedt HW, Sickert M, Köstler H (2000). Diagnostic value of the fast-FLAIR sequence in MR imaging of intracranial tumors. Eur Radiol.

[25] Eidel O, Neumann J-O, Burth S (2016). Automatic Analysis of Cellularity in Glioblastoma and correlation with ADC Using Trajectory Analysis and automatic nuclei counting. PLoS ONE.

[26] Bulik M, Jancalek R, Vanicek J (2013). Potential of MR spectroscopy for assessment of glioma grading. Clin Neurol Neurosurg.

[27] Horská A, Barker PB (2010). Imaging of Brain tumors: MR Spectroscopy and metabolic imaging. Neuroimaging Clin.

[28] Harris RJ, Cloughesy TF, Liau LM (2015). pH-weighted molecular imaging of gliomas using amine chemical exchange saturation transfer MRI. Neuro Oncol.

[29] Yao J, Tan CHP, Schlossman J (2019). pH-weighted amine chemical exchange saturation transfer echoplanar imaging (CEST-EPI) as a potential early biomarker for bevacizumab failure in recurrent glioblastoma. J Neurooncol.

[30] Chang PD, Malone HR, Bowden SG (2017). A multiparametric model for Mapping Cellularity in Glioblastoma using Radiographically localized biopsies. AJNR Am J Neuroradiol.

[31] Gates EDH, Lin JS, Weinberg JS (2019). Guiding the first biopsy in glioma patients using estimated Ki-67 maps derived from MRI: conventional versus advanced imaging. Neuro Oncol.

[32] Bobholz SA, Lowman AK, Connelly JM et al (2022) Non-invasive tumor probability maps developed using autopsy tissue identify novel areas of tumor beyond the imaging-defined margin. 10.1101/2022.08.17.22278910. medRxiv

[33] Louis DN, Perry A, Wesseling P (2021). The 2021 WHO classification of tumors of the Central Nervous System: a summary. Neuro Oncol.

[34] Ashburner J, Friston KJ (2000). Voxel-based Morphometry—the methods. NeuroImage.

[35] Maes F, Collignon A, Vandermeulen D (1997). Multimodality image registration by maximization of mutual information. IEEE Trans Med Imaging.

[36] Bobholz SA, Lowman AK, Barrington A (2020). Radiomic features of Multiparametric MRI Present stable associations with analogous histological features in patients with Brain Cancer. Tomography.

[37] McGarry SD, Hurrell SL, Iczkowski KA (2018). Radio-pathomic maps of epithelium and Lumen Density predict the location of high-Grade prostate Cancer. Int J Radiat Oncol Biol Phys.

[38] Duenweg SR, Fang X, Bobholz SA (2021). Diffusion restriction comparison between Gleason 4 fused glands and cribriform glands within patient using whole- mount prostate pathology as ground truth. Tomography.

[39] Chen X, Zhang M, Gan H (2018). A novel enhancer regulates MGMT expression and promotes temozolomide resistance in glioblastoma. Nat Commun.

[40] Kitange GJ, Carlson BL, Schroeder MA (2009). Induction of MGMT expression is associated with temozolomide resistance in glioblastoma xenografts. Neuro Oncol.

