# Correction to: Radio-pathomic maps of glioblastoma identify phenotypes of non-enhancing tumor infiltration associated with bevacizumab treatment response

**DOI:** 10.1007/s11060-024-04641-2

**Published:** 2024-03-18

**Authors:** Samuel A. Bobholz, Alisha Hoefs, Jordyn Hamburger, Allison K. Lowman, Aleksandra Winiarz, Savannah R. Duenweg, Fitzgerald Kyereme, Jennifer Connelly, Dylan Coss, Max Krucoff, Anjishnu Banerjee, Peter S. LaViolette

**Affiliations:** 1 Department of Radiology, Medical College of Wisconsin, 8701 Watertown Plank Rd, 53226 Milwaukee, WI USA; 2 Department of Biophysics, Medical College of Wisconsin, Milwaukee, WI USA; 3 Department of Neurology, Medical College of Wisconsin, Milwaukee, WI USA; 4 Department of Pathology, Medical College of Wisconsin, Milwaukee, WI USA; 5 Department of Neurosurgery, Medical College of Wisconsin, Milwaukee, WI USA; 6 Department of Biostatistics, Medical College of Wisconsin, Milwaukee, WI USA; 7 Department of Biomedical Engineering, Medical College of Wisconsin, Milwaukee, WI USA

**Journal of Neuro-Oncology**

10.1007/s11060-024-04593-7

In Fig. 4 of this article the blue and red lines were incorrectly labeled. The blue ones should have been labeled as ‘Yes’ and the red ones as ‘No’ (instead of the other way around).

The original article has been corrected.

